# Comparison of poly-3-hydroxybutyrate (P3HB) synthesis by *Bacillus cereus* and *Azotobacter vinelandii* OP: effect of agitation on the accumulation and physicochemical properties of the biopolymer

**DOI:** 10.1186/s40643-025-00978-2

**Published:** 2025-12-20

**Authors:** Isabo Morales-Núñez, Marcela Cancino, Eric Pérez, Ricardo I. Castro, Maribel Mamani, Howard Ramírez-Malule, Álvaro Díaz-Barrera, Rodrigo Andler

**Affiliations:** 1 Present Address: Escuela de Ingeniería en Biotecnología, Centro de Biotecnología de los Recursos Naturales (CENBio), Universidad Católica del Maule, Talca, Chile; 2 Multidisciplinary Agroindustry Research Laboratory, Carrera de Ingeniería en Construcción, Instituto de Ciencias Químicas Aplicadas, Universidad Autónoma de Chile, Talca, Chile; 3 Laboratorio de Bioprocesos, Centro de Biotecnología de los Recursos Naturales (CENBio), Universidad Católica del Maule, Talca, Chile; 4 School of Chemical Engineering, Universidad del Valle, 760042 Cali, Colombia; 5 Escuela de Ingeniería Bioquímica, Pontificia Universidad Católica de Valparaíso, Valparaíso, Chile

**Keywords:** *Azotobacter vinelandii* OP, Agitation rate, *Bacillus cereus*, k_L_a, P3HB, Poly-3-hydroxybutyrate

## Abstract

Poly-3-hydroxybutyrate (P3HB) is a biodegradable thermoplastic polyester with mechanical and thermal properties comparable to those of petrochemical-based plastics. In this study, the synthesis of P3HB by *Bacillus cereus* ATCC 14579 and *Azotobacter vinelandii* OP ATCC 13705 in complex media under different agitation conditions and cultivation times was evaluated. The growth kinetics of each microorganism responded differently to changes in agitation patterns. Maximum cell concentrations of 2.4 g L^−1^ and 4.3 g L^−1^ were achieved at 200 rpm (24 h) for *B. cereus* and 150 rpm (48 h) for *A. vinelandii*, respectively. While *B. cereus* reached an accumulation of 31.3% (0.37 g P3HB L^−1^), *A. vinelandii* OP achieved 55.8% (2.3 g P3HB L^−1^). The biopolymer was characterized by ATR-FTIR, with a prominent carbonyl (C = O) stretching vibration observed at 1724 cm^−1^. SEC-HPLC analysis revealed mean molecular weights (MMW) weights of 80,050 g mol^−1^ to 116,960 g mol^−1^ for *B. cereus* and from 75,805 to 111,000 g mol^−1^ for *A. vinelandii* OP. TGA/DSC analysis revealed that higher agitation rates decrease crystallinity and thermal stability by altering polymer chain alignment. The volumetric oxygen transfer coefficient (k_L_a) confirmed the role of oxygen availability on P3HB. These results highlight two promising strains with distinct metabolic behaviors and strong potential for scale-up in P3HB production.

**Graphical abstract:**

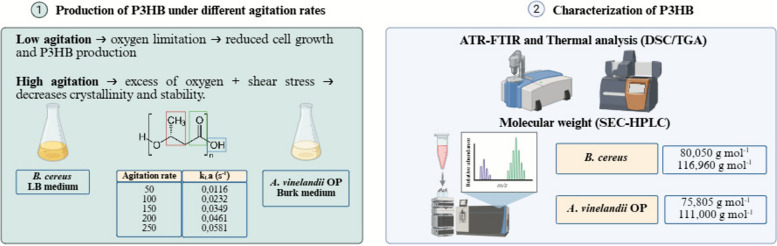

## Introduction

Poly-3-hydroxybutyrate (P3HB) is a biodegradable thermoplastic polyester belonging to the family of polyhydroxyalkanoates (PHA), whose mechanical and thermal properties resemble those of conventional petrochemical-based plastics (Kanjanachumpol et al. 2013). Various microorganisms synthesize and store it as insoluble intracellular granules, serving as an energy reserve when carbon sources are abundant but essential nutrients—such as nitrogen, phosphorus, oxygen, or other elements—are limited (San Miguel-González et al. 2024). P3HB production begins with the precursor acetyl-CoA and is mediated by the enzymes beta-ketothiolase (*phaA*), acetoacetyl-CoA reductase (*phaB*), and PHA synthase (*phaC*), encoded within an operon that is activated under environmental stress. In such conditions, the cell diverts acetyl-CoA from the Krebs cycle toward the synthesis and accumulation of P3HB (Martínez et al. 2023). Molecular weight can range from 100 to 1,000 kDa in native strains and is a key factor influencing both mechanical properties and biodegradability. Higher molecular weights enhance the biopolymer’s strength and toughness, while lower values promote degradation and reduce thermal stability (Agus et al. 2006).

More than 300 microbial species capable of producing and storing this biopolymer have been identified (Peña et al. 2014). Among them, two interesting bacteria are described: i) *Bacillus cereus*, a Gram-positive, facultative anaerobe widely distributed in the environment and notable for its resistance to extreme temperature and pH variations (Ellouze et al. 2021), and ii) *Azotobacter vinelandii*, a Gram-negative, strictly aerobic bacterium commonly found in neutral to alkaline soils, which also produces alginate—a polysaccharide of considerable biotechnological importance (Campos et al. 2020). However, compared with *Cupriavidus necator*, the model microorganism for P3HB production (De Mello et al. 2023), studies on *B. cereus* and *A. vinelandii* remain limited to flask scale and evaluating the effect of stirring speed. This gap highlights the need to investigate operational factors—such as oxygen availability—that affect P3HB synthesis in different microbial systems. Notably, *A. vinelandii* OP, a mutant that does not synthesize alginate, exhibits a high respiration rate, influencing biopolymer production efficiency under varying oxygen transfer conditions (Segura et al. 2009). However, the effect of respiratory rate on this production has not yet been studied in *B. cereus.*

The availability of oxygen in the liquid medium is governed by two fundamental processes: the rate of oxygen transfer from the gas phase to the medium, and the rate of oxygen consumption by the cells (Garcia-Ochoa and Gomez 2009). The rate of agitation directly affects oxygen transfer, which in turn impacts metabolic pathways and enzymatic activities involved in P3HB synthesis. This can alter the redox balance of cofactors like NADH/NAD⁺, leading to an accumulation or reduction of reducing power (García et al. 2018). Yet, excessive or insufficient stirring (leading to high or low shear forces) may induce cellular stress and disrupt polymerization, causing fluctuations in the molecular weight of the biopolymer. The fundamental difference in the metabolism of *B. cereus* and *A. vinelandii* OP lies in how each microorganism physiologically responds to varying environmental scenarios, activating regulatory mechanisms according to their nutritional needs (Hamdy et al. 2022).

A key parameter to assess oxygen transfer efficiency is the volumetric oxygen transfer coefficient (k_L_a), which depends on factors such as agitation speed, orbital diameter, and filling volume (Heyman et al. 2019). A high k_L_a enhances respiration and enzymatic activity, while a low k_L_a restricts them. However, in shake flask systems, k_L_a is constrained by vessel geometry and the type of closure, necessitating experimental determination in each case. Despite their simplicity, flasks allow for the identification of kinetic trends in P3HB growth and production, serving as a basis for process optimization and scale-up to bioreactors (Heyman et al. 2019).

Although previous work has independently examined P3HB production in *Bacillus* or *Azotobacter* species, no studies have systematically compared these two physiologically distinct microorganisms under controlled agitation conditions at the flask scale. Furthermore, the application of the correlation for estimating oxygen transfer (k_L_a) has not been previously reported for these bacteria. By integrating kinetic, physicochemical, and thermomechanical analyses, this work provides the first comparative assessment of how oxygen transfer and agitation influence P3HB synthesis and polymer properties.

In this study, P3HB production by *B. cereus* ATCC 14579 and *A. vinelandii* OP ATCC 13705 was evaluated in complex media under varying agitation conditions and culture durations. Additionally, we analyzed how agitation rates influence the biopolymer’s properties for potential applications. Optimizing bioprocess conditions enables the production of P3HB with tailored characteristics, which is key to positioning these biomaterials competitively in the market.

## Materials and methods

### Microorganisms and media composition

The microorganisms used in this study were *B. cereus* ATCC 14579 and *A. vinelandii* OP ATCC 13705, both cryopreserved at − 40 °C. *Bacillus cereus* was cultured in LB (Luria–Bertani) medium, while *A. vinelandii* OP was grown in Burk medium with the following composition (g L^−1^): 0.66 K_2_HPO_4_, 0.16 KH_2_PO_4_, 1 (NH_4_)_2_SO_4_, 0.056 CaSO_4_·2H_2_O, 0.2 NaCl, 0.2 MgSO_4_·7H_2_O, 0.0029 Na_2_MoO_4_·2H_2_O, 0.027 FeSO_4_·7H_2_O, 20 sucrose, and 5 yeast extract. To prevent salt precipitation during sterilization, the components CaSO_4_·2H_2_O, NaCl, MgSO_4_·7H_2_O, Na_2_MoO_4_·2H_2_O and FeSO_4_·7H_2_O of the Burk medium was sterilized separately. Afterward, the pH was adjusted to 7.2 using 4 M NaOH.

### Cultivation conditions

The inoculum of *B. cereus* ATCC 14579 and *A. vinelandii* OP ATCC 13705 was prepared in 100-mL shake flasks containing 20 mL of culture medium, inoculated at 5% (v/v), and incubated at 30 °C on an orbital shaker (JSR, JSSI-100 C) at 100 rpm. The incubation time was 24 h for *B. cereus* and 72 h for *A. vinelandii* OP. Primary cultures were carried out in 250-mL flasks containing 50 mL of culture medium, inoculated at 2% (v/v). For *B. cereus*, cultures were incubated for 24 and 48 h, while for *A. vinelandii* OP, incubation lasted between 48 and 72 h. At the end of each cultivation period, the pH of the supernatant was measured. The cultivation times were determined based on the growth kinetics and metabolic states of each microorganism.

### Recovery of P3HB

To extract and purify P3HB from the cultures, the protocol described by Heinrich et al. (2012) was followed. A total of 0.1 g of cell biomass was transferred into 5 mL of 10% (v/v) sodium hypochlorite (NaClO) and incubated for 1 h at room temperature. Then, 2.5 mL of distilled water was added, and the mixture was incubated for 8 h on an orbital shaker. After incubation, the sample was centrifuged for 10 min at 4,000 × *g*, the supernatant was discarded, and distilled water was added again. The sample was centrifuged once more under the same conditions, after which 5 mL of isopropanol was added. The supernatant was discarded, and the sample was placed in an oven at 80 °C for 24 h.

### Attenuated total reflectance fourier transform infrared spectroscopy (ATR-FTIR)

The surface structure of the recovered P3HB was evaluated using ATR-FTIR (Specac Quest, Spectrum Two). Transmittance was measured over a range of 400–4000 cm^−1^ with a resolution of 4 cm^−1^. The results were compared to those of pure P3HB control samples using OMNIC software, version 9.2.86.

### Thermogravimetric analysis (TGA)

The P3HB samples were analyzed by TGA to assess thermal stability. Each 5-mg sample was homogenized using a mortar and pestle (Parra-Palma et al. 2024). A Discovery SDT-650 thermogravimetric analyzer (TA Instruments, New Castle, DE, USA) was used to evaluate the chemical properties associated with the degradation process. Dry samples were heated in the presence of air as a reactive gas at a constant rate of 10 °C min^−1^, reaching between 50 °C and 600 °C, with an airflow rate of 50 mL min^−1^. Additionally, the electronic balance was protected by a nitrogen flow of 50 mL min^−1^.

### Differential scanning calorimetry (DSC)

DSC was used to further characterize the P3HB samples. After placing the samples into α-Al_2_O_3_ crucibles, they were analyzed using an SDT-Q600 simultaneous DSC–TGA analyzer (TA Instruments). The analysis was performed from room temperature (25 °C) up to 600 °C. Following a 1-min equilibration at 25 °C, the samples were heated at a constant rate of 10 °C min^−1^. Sapphire was used as the reference material, and the equipment was calibrated according to the manufacturer’s instructions. The TRIOS TA-Instrument Thermal Analysis System Program was used to calculate the transition enthalpy (ΔH, in J/g), onset temperature (To), peak temperature (Tp), and conclusion temperature (Tc).

### High-performance liquid chromatography size exclusion chromatography (SEC-HPLC)

The mean molecular weight (MMW) of P3HB was determined using SEC-HPLC (YL9100 Plus, YOUNG IN Chromass). P3HB was first dissolved in chloroform at a concentration of 5 mg mL^−1^ and left to stand at room temperature for 24 h. The mobile phase was prepared by mixing tetrahydrofuran and acetic acid in a 95:5 ratio. A PSS GPC/SEC column (3 μm, 8 × 300 mm) was used, with a flow rate of 0.3 mL min^−1^ and a total run time of 45 min.

### Confocal microscopy

For the analysis of P3HB using confocal microscopy, a protocol adapted from Juengert et al. (2018) was followed. Nile Red reagent was diluted in HPLC-grade acetone to a final concentration of 10 μg mL^−1^. A 1 mL sample of broth culture was taken at 30 °C, and biomass was recovered by centrifugation in 1.5 mL microcentrifuge tubes at 5,000 × *g* for 15 min. The supernatant was discarded, and 200 μL of the Nile Red solution was added to the pellet, followed by mixing for 1 min. Next, 20 μL of the stained suspension was placed on a microscope slide pre-coated with 1% (w v^−1^) agarose gel and covered with a coverslip. The sample was examined using a confocal microscope (Leica, Stellaris 5) at 100 × magnification with a digital zoom of 2. Excitation and emission wavelengths were set to 590 nm and 560 nm, respectively.

### Volumetric mass transfer coefficient measurements

The k_L_a at flask scale was calculated using the correlation described by Klöckner and Büchs (Klöckner and Büchs 2012), expressed as:

1 $${k}_{L}a=\text{0,5}\times {d}^\frac{73}{36}\times n\times {d}_{0}^\frac{1}{4}\times {V}_{L}^{-\frac{8}{9}}\times {D}^\frac{1}{2}\times {v }^{-\frac{13}{54}} \times {g}^{-\frac{7}{54}}$$

Where d is the maximum internal diameter of the flask (m), n is the shaking speed (s^−1^), ${d}_{0}$ is the shaker rotation diameter (m), ${V}_{L}$ is the working volume of the flask (${m}^{3}$), D is the diffusion coefficient (${m}^{2}$ s^−1^), v is the kinematic viscosity (${m}^{2}$ s^−1^) and g the gravitational acceleration (${m/s}^{2}$).

#### Analytical determinations

Biomass quantification was performed by gravimetric dry weight analysis. For this, 40 mL of culture was collected at the final point. Samples were centrifuged in pre-dried tubes (80 °C, 20 min) at 4,000 × *g*. The supernatant was discarded, and the resulting cell pellet was resuspended in 10 mL of 0.9% (w/v) NaCl. This washing step was repeated twice. Finally, the cell pellets were dried at 80 °C for 24 h, cooled in a desiccator, and weighed on an analytical balance.

#### General calculations

The percentage P3HB accumulation (%), volumetric productivity (Q_P3HB_), and specific productivity (q_P3HB_) were calculated using the following formulas:

2 $$Accumulation P3HB=\frac{Dry weigth of P3HB \left(g\right)}{Dry biomass \left(g\right)}x 100$$

3 $${Q}_{P3HB}=\frac{{P}_{1}-{P}_{0}}{tV}$$

4 $${q}_{P3HB}=\frac{{P}_{1}-{P}_{0}}{t({x}_{1}-{x}_{0})}$$

where ${P}_{1}$ is the final mass obtained, which is P3HB (g), ${P}_{0}$ is the initial mass of P3HB (g), t is the culture time (h) y V is the culture volume (L), ${x}_{1}$ is the final biomass concentration (g L^−1^) and ${x}_{0}$ is the initial biomass concentration (g L^−1^).

## Results and discussion

### Cell growth and P3HB production

**Fig. 1:**
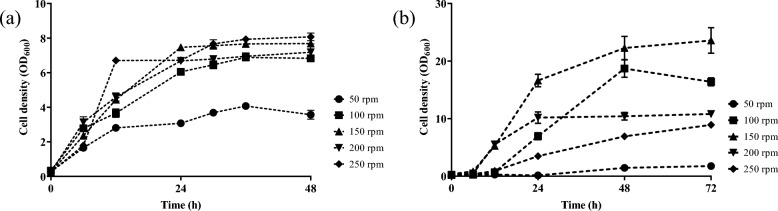
Growth kinetics of ** a** *Bacillus cereus* and ** b** *Azotobacter vinelandii* OP under different shaking speeds: (●) 50 rpm, (■) 100 rpm, (▲) 150 rpm, (▼) 200 rpm, and (♦) 250 rpm. Data represents the mean of triplicate samples ± standard error

Cell growth of *B. cereus* and *A. vinelandii* OP (Fig. 1) was evaluated under different agitation rates and culture durations. In *B. cereus* (Fig. 1a), up to 24 h of culture, greater growth is observed at a stirring speed of 150 rpm, reaching a cell concentration of 7.46 ± 0.02, and at 48 h of culture at a stirring speed of 250 rpm, it reached 8.04 ± 0.07 It is also observed that, at approximately 28 h of culture, the stationary phase begins in most of the conditions evaluated. On the other hand, *A. vinelandii* OP (Fig. 1b) shows a more prolonged growth pattern, where at 48 h greater growth was obtained at a stirring speed of 150 rpm, reaching 22.27 ± 0.67, and at 72 h, 23.57 ± 0.74. *B. cereus* exhibits faster growth, while *A. vinelandii* OP reaches higher cell densities with a longer exponential phase and a delayed stationary phase. This comparison is essential for subsequently interpreting how each microorganism responds to agitation and oxygen transfer conditions in relation to P3HB synthesis.

**Fig. 2:**
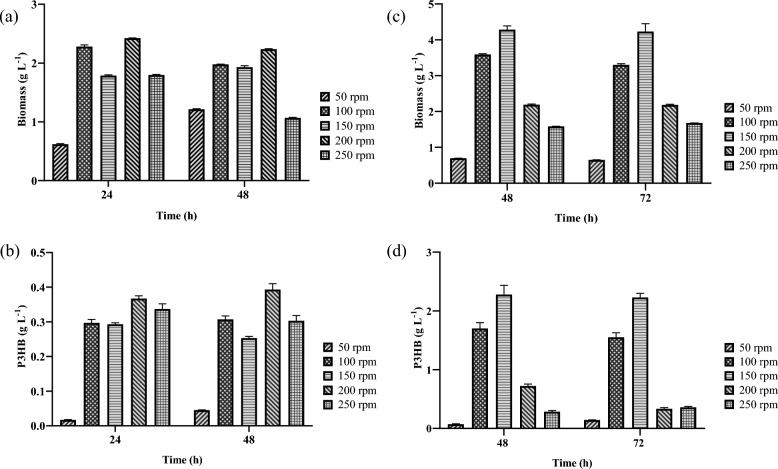
Effect of agitation speed and culture time on biomass concentration (g L^−1^) and P3HB concentration (g L^−1^): ** a**, ** b** *Bacillus cereus*; ** c**, ** d** *Azotobacter vinelandii* OP. Bars represent mean ± standard deviation (n = 3). Different letters indicate statistically significant differences (*p* < 0.05) between treatments as determined by ANOVA followed by Tukey’s multiple comparisons test

In cultures of *B. cereus*, the highest biomass concentration (Fig. 2a) was obtained at a shaking speed of 200 rpm after 24 h, reaching 2.42 ± 0.01 g L^−1^. By contrast, at 50 rpm, biomass production was significantly lower at both time points—0.619 ± 0,01 g L^−1^ at 24 h and 1.21 ± 0,01 g L^−1^ at 48 h—indicating that low agitation creates unfavorable conditions for microbial growth. Concerning pH, alkalinization of the medium was observed under all tested conditions. The initial pH of 6.87 increased to 9.09 ± 0.01 by the end of the culture period. This shift is attributed to the nitrogen metabolism of *B. cereus*, which utilizes amino acids from the LB medium as sources of both nitrogen and carbon. During this process, amino acids undergo oxidative deamination, producing α-keto acids and ammonia (NH₃) as by-products. The released ammonia dissolves in the medium, (Kasu et al. 2024), which in turn leads to the release of hydroxyl ions (OH −) and a consequent rise in pH.

In *A. vinelandii* OP cultures, the highest biomass concentration (Fig. 2c) was achieved at 48 h with an agitation speed of 150 rpm, reaching 4.28 ± 0,10 g L^−1^. At 72 h, the biomass remained similar, with a value of 4.23 ± 0,21 g L^−1^. By contrast, the lowest values were recorded at 50 rpm, indicating that low agitation significantly affects cell growth in this bacterium. Regarding pH behavior, *A. vinelandii* OP cultures exhibited a distinct pattern compared to *B. cereus*. Starting from an initial pH of 7.2 ± 0,01, a progressive decrease was observed under all tested conditions, with a notable drop to 4.71 ± 0.01 at 50 rpm. This acidification of the Burk medium negatively affects the microorganism’s ability to channel excess carbon into P3HB synthesis, leading to metabolic imbalance (Bher et al. 2022). (Sashidhar and Podile 2009). Excess carbon is diverted to alternative pathways, resulting in the production and excretion of organic acids—primarily acetic and succinic, and to a lesser extent, lactic acid—which contribute to the acidification of the medium.

The highest P3HB concentration in *B. cereus* (Fig. 2b) was obtained at 24 h with an agitation speed of 200 rpm, reaching 0.36 ± 0.01 g L^−1^. As with biomass, P3HB production was lowest at 50 rpm, with only 0.02 ± 0.01 g L^−1^ at 24 h and 0.04 ± 0.01 g L^−1^ at 48 h. These results indicate that 200 rpm provides more favorable conditions for both biomass growth and P3HB accumulation. It has been reported that extending cultivation times even further can decrease P3HB of production. This trend has been reported in other studies; for example, Hamdy et al. (2022) observed that P3HB content in *B. cereus* SH-02 increased from 0.79 ± 0.03 g L^−1^ at 24 h to 2.15 ± 0.12 g L^−1^ at 72 h. However, production declined after that point, as the bacteria began to utilize the accumulated P3HB as a nutrient source (Hamdy et al. 2022).

The highest amount of P3HB in *A. vinelandii* OP cultures (Fig. 2d) was obtained at an agitation speed of 150 rpm, reaching 2.27 ± 0.15 g L^−1^ at 48 h and 2.22 ± 0.07 g L^−1^ at 72 h. These values were considerably higher than those observed under the other conditions tested. At 50 rpm, P3HB production was minimal, with only 0.07 ± 0.01 g L^−1^ at 48 h and 0.14 ± 0.01 g L^−1^ at 72 h. This result may be attributed to the fact that *A. vinelandii* OP is a strictly aerobic microorganism with a high respiration rate (Setubal et al. 2009). As reported by (Wu et al. 2000), this strain possesses highly efficient respiratory regulatory mechanisms that allow it to adapt to microaerophilic conditions. However, as observed in this study, agitation at 50 rpm did not provide the necessary oxygen transfer conditions to support adequate growth or P3HB production in *A. vinelandii* OP.

At low agitation speeds, respiratory and metabolic demands are not fully met, leading to intracellular imbalance due to the accumulation of acetyl-CoA and reducing equivalents. This favors the redirection of metabolism toward P3HB biosynthesis, which functions both as an energy storage mechanism and an electron sink (Wu et al. 2000; Natzke et al. 2018). This behavior may correspond to what was observed at 100 rpm in this study. Under high-aeration conditions, such as those at 250 rpm, oxygen availability may exceed the bacterium’s metabolic requirements. In response, the regulatory system redirects the electron flow through the respiratory chain to dissipate excess cytoplasmic oxygen and prevent the overproduction of reactive oxygen species. Concurrently, the pentose phosphate pathway becomes the primary source of NADPH, which meets the energy demand associated with increased metabolic flux and contributes to the protection of oxidation-sensitive enzymes, thereby helping to maintain intracellular redox balance (Natzke et al. 2018; Castillo et al. 2020).

In relation to the percentage of P3HB accumulation, *B. cereus* reached a maximum of 31.28% ± 1.74 at a stirring speed of 250 rpm and 24 h of culture, while at low stirring speeds, percentages close to 6.12% ± 0.30 were recorded at 48 h. In contrast, *A. vinelandii* OP showed accumulations with a maximum of 55.84% ± 0.69 at a stirring speed of 100 rpm and 48 h of culture, followed by 52.96% ± 0.98 at 150 rpm at 72 h of culture. The slight decrease in P3HB accumulation over time may be attributed to partial consumption of the biopolymer as a carbon and energy source in response to nutrient depletion in the medium—a behavior also reported in other studies involving nitrogen-fixing bacteria under nutritional stress conditions (Yoneyama et al. 2015; Portugal-Nunes et al. 2017; Velázquez-Sánchez et al. 2020).

The volumetric productivity of both microorganisms was also evaluated under the different culture conditions (Table 1). In *B. cereus* cultures, the highest volumetric productivity of P3HB was observed at 200 rpm after 24 h, reaching 15.30 ± 0.20 mg L^−1^ h^−1^. By contrast, the highest specific productivity was achieved at 250 rpm after 24 h, with a value of 14.10 ± 0.20 mg g^−1^ h^−1^. In *A. vinelandii* OP cultures, the highest volumetric productivity was recorded at 150 rpm after 48 h, reaching 47.46 ± 3.20 mg L^−1^ h^−1^, along with a specific productivity of 11.01 ± 0.51 mg g^−1^ h^−1^. These results suggest that although *B. cereus* grows more rapidly during the early stages of cultivation—favoring initial P3HB accumulation—*A. vinelandii* OP demonstrates superior metabolic efficiency and productive capacity overall. Currently, there are no studies specifically addressing the effects of agitation rate or oxygen transfer on P3HB production in *B. cereus* submerged cultures. However, various studies have examined the use of alternative carbon sources, highlighting the organism’s capacity to metabolize a wide range of substrates. This metabolic versatility, combined with its short culture times, positions *B. cereus* as a promising candidate for the valorization of low-cost carbon sources, such as agro-industrial wastes (Martínez-Herrera et al. 2023).

**Table 1:** A. Kinetic parameter calculations for *Bacillus cereus* under different agitation rates and culture times. B. Kinetic parameter calculations for *Azotobacter vinelandii* OP under different agitation rates and culture times

A	*Bacillus cereus*			
Agitation rate (rpm)		Time (h)	Q_PHB_ (mg L^−1^ h^−1^)	q_PHB_ (mg g^−1^ h^−1^)
50		24	1.11 ± 0.12	0.05 ± 0.04
		48	1.03 ± 0.04	0.03 ± 0.01
100		24	12.42 ± 0.30	9.07 ± 0.30
		48	6.38 ± 0.20	5.38 ± 0.20
150		24	12.19 ± 0.10	11.35 ± 0.10
		48	5.34 ± 0.10	4.61 ± 0.10
200		24	15.30 ± 0.20	10.50 ± 0.20
		48	7.04 ± 0.20	5.25 ± 0.20
250		24	14.10 ± 0.20	13.04 ± 0.20
		48	6.30 ± 0.10	5.90 ± 0.10

B	*Azotobacter vinelandii* OP			
Agitation rate (rpm)		Time (h)	Q_PHB_ (mg L^−1^ h^−1^)	q_PHB_ (mg g^−1^ h^−1^)
50		48	1.18 ± 0.35	1.69 ± 0.52
		72	1.56 ± 0.08	2.41 ± 0.18
100		48	41.43 ± 2.60	0.58 ± 0.20
		72	31.23 ± 1.80	0.17 ± 0.06
150		48	47.46 ± 3.20	11.01 ± 0.51
		72	30.95 ± 0.90	7.35 ± 0.14
200		48	15.01 ± 0.69	3.78 ± 0.09
		72	5.74 ± 0.36	2.96 ± 0.09
250		48	6.23 ± 0.05	4.33 ± 0.23
		72	7.43 ± 0.08	4.46 ± 0.05

For *A. vinelandii* OP, the effect of agitation rate on P3HB production has not been evaluated at the shake flask scale; however, it has been studied at the bioreactor scale. The only reported precedent under similar conditions corresponds to the study by (Urtuvia et al. 2020), cultures were conducted to evaluate the production of poly-3-hydroxybutyrate-co-valerate (P3HBV) in the presence of different precursors of the 3HV fraction of the bioplastic, using various stirring speeds. In flask-scale cultures, agitation was maintained at 200 rpm for 64 h, resulting in a biomass concentration of 3.4 ± 0,50 g L^−1^ and an accumulation percentage of 41.8% ± 1,40 (w/w). By contrast, in bioreactor-scale cultures where precursors were added to the medium, the highest P3HB accumulation was achieved with hexanoate, yielding 3.5 ± 0,10 g L^−1^ of biomass and 2.1 ± 0,10 g L^−1^ of P3HB, corresponding to an accumulation percentage of 59.3% ± 2.0 (w/w) (Urtuvia et al. 2020).

In *B. cereus*, P3HB synthesis has been proposed to be linked to stress response regulatory systems. The Spo0A protein—traditionally associated with sporulation—has been reported to regulate genes involved in P3HB biosynthesis in related *Bacillus* species (Chen et al. 2010); (Li et al. 2020). Similarly, systems such as ResDE may influence this process by modulating redox metabolism under oxygen-limiting conditions (Zhou et al. 2018). While our study did not directly assess these molecular mechanisms, the observed dependence of P3HB accumulation on agitation rate may reflect such regulatory responses to oxygen availability.

In *A. vinelandii*, several pathways—including PEP-PTS, phbR, and the Gac/Rsm system—have been described as regulators of P3HB synthesis (Mitra et al. 2022). The patterns observed here could be consistent with these known control circuits; however, this interpretation remains hypothetical since our study focused on phenotypic and physicochemical parameters. Future transcriptomic or proteomic analyses could help to confirm whether these pathways are indeed involved under the agitation conditions tested.

### Effect of k_L_a on the biomass concentration and the production and MMW of P3HB

The effect of agitation rates on growth and metabolite production has been studied in various microbial systems. In this work, the volumetric oxygen transfer coefficient (k_L_a) was estimated theoretically using the correlation proposed by Klöckner and Büchs (2012) to provide a comparative indicator of oxygen transfer potential under different agitation rates. It is important to clarify that these values were not experimentally measured and therefore represent relative rather than absolute oxygen transfer capacities. Consequently, the analysis presented below focuses on the trends linking agitation intensity, estimated oxygen transfer potential, and P3HB accumulation across the two strains.

There are no reports of Klöckner et al. (2013) correlation being applied to estimate k_L_a in flask-scale cultures of *B. cereus* or *A. vinelandii* OP, or in other bacteria. Therefore, this study proposes its use as a first approximation of oxygen transfer behavior in these systems. Table 2 shows the values estimated using this correlation, together with the k_L_a values previously determined by gassing out in the bacteria studied. Using the correlation, values ranging from 83.5 s^−1^ to 209.6 s^−1^ were obtained, following the expected trend of increasing with agitation speed. It should be noted that various models and correlations have been developed to theoretically estimate k_L_a in bioreactors, which can be used to compare different scales within geometrically similar systems, such as culture flasks (Klöckner and Büchs 2012). However, the estimated values typically show an accuracy of approximately ± 30% (Klöckner et al. 2013) and may lead to overestimation—even under controlled conditions—because of the influence of variables such as flask geometry, filling volume, and shaking amplitude. Nonetheless, these calculations provide a useful initial approximation of the system’s oxygen transfer behavior.

**Table 2:** Estimated k_L_a values under different stirring speeds for the cultures studied

Microorganism	Medium	Cultivation mode	Agitation rate (rpm)	Working volume (L)	k_L_a (h ^−1^)	References
*Bacillus thuriengensis* GP139	Molasses and soy Flour	Shake flasks	250	0.1	37.1	Salazar-Magallón et al. (2019)
		Bioreactor	250	4	11.16	
			650	4	38.5	
			1000	4	75.6	
*Bacillus megaterium* DSM 32 T	Mineral medium	Bioreactor	100	4	7.2	Faccin et al. (2013)
			200	4	21.6	
			300	4	46.8	
			400	4	64.8	
			500	4	97.2	
			600	4	133.2	
*Azotobacter vinelandii* AT9	Burk-Sacarose	Bioreactor	200	2	11.5	Medina et al. (2023)
			370	2	39.0	
			500	2	68.0	
*Azotobacter vinelandii* ATCC 9046	Burk-Sacarose	Bioreactor	260	2	22.3	Diaz-Barrera et al. (2009)
			300	2	39.0	
			340	2	47.2	
			400	2	56.5	
			450	2	63.2	
			500	2	65.5	
*Azotobacter vinelandii* ATCC 9046	Burk-Sacarose	Bioreactor	140	1	8.69	Barrales-Cureño et al. (2017)
			180	1	12.67	
			200	1	14.5	
			340	1	28.6	
*Bacillus cereus* ATCC 14579 and *Azotobacter vinelandii* ATCC 13705	LB-Burk sacarose	Shake flasks	50	0.05	41.73	This study
			100	0.05	83.5	
			150	0.05	125.7	
			200	0.05	165.9	
			250	0.05	209.6	

Although a higher k_L_a increases the availability of dissolved oxygen, it does not always correlate with increased biomass or P3HB production. Under high-transfer conditions, mechanical stress from agitation can damage bacterial cells, potentially limiting growth and viability (Moradkhani et al. 2017).

**Fig. 3:**
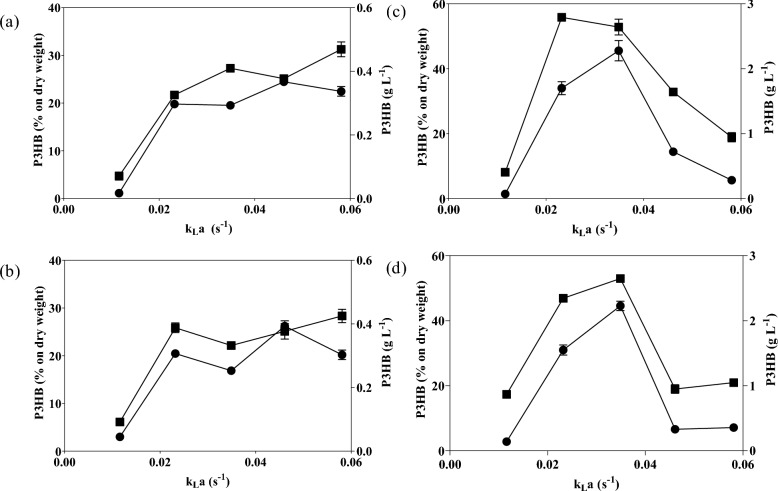
Relationship between oxygen transfer coefficient (k_L_a), percent P3HB accumulation, and P3HB concentration (g L^−1^) under different agitation rates: (●) P3HB concentration (g L^−1^); (■) P3HB accumulation percentage. **a**,** b** *Bacillus cereus* 24 and 48 h; **c**,** d** *Azotobacter vinelandii* OP 48 and 72 h

*B. cereus* can grow under both aerobic and anaerobic conditions, which gives it high metabolic flexibility and adaptability to different aeration levels, as reflected in its P3HB production and accumulation percentages. By contrast, *A. vinelandii* OP is a strict aerobe that requires oxygen for growth. However, it has evolved highly efficient respiratory mechanisms that protect oxygen-sensitive enzymes, such as nitrogenase, from oxidative stress by tightly regulating its intracellular environment through high rates of oxygen consumption. These physiological differences explain the greater tolerance of *B. cereus* to varying agitation conditions and the high P3HB accumulation capacity of *A. vinelandii* OP under intermediate oxygenation conditions. The Fig. 3 shows how the accumulation of P3HB in *A. vinelandii* OP correlates closely with the increase in k_L_a, unlike *B. cereus*, which shows a more stable response that is less dependent on oxygen availability. It provides a preliminary framework for selecting oxygenation conditions in future scaling studies, especially for *A. vinelandii* OP, where small variations in k_L_a produce significant changes in yield.

This analysis provides a useful framework for understanding relative oxygenation behavior in both species, particularly in *A. vinelandii* OP, where small variations in estimated k_L_a correspond to notable changes in the P3HB production yield. However, these results should be interpreted as indicative comparative trends rather than quantitative k_L_a targets for process design or scale-up applications.

### Characterization of P3HB

#### Physicochemical and thermomechanical analysis of P3HB

**Fig. 4:**
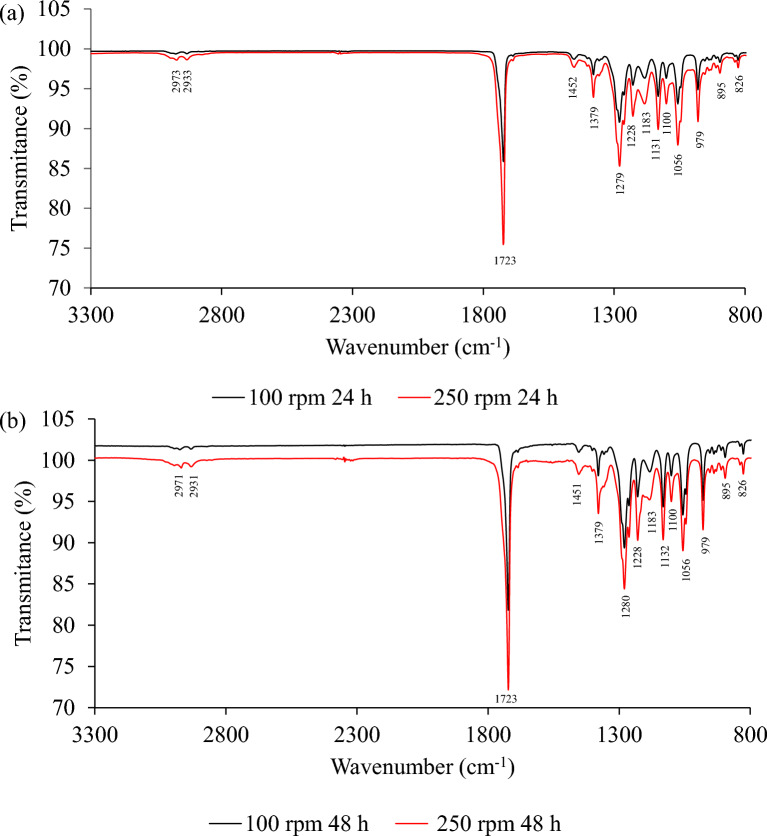
ATR-FTIR spectra of P3HB extracted from ** a** *Bacillus cereus* and ** b** *Azotobacter vinelandii* OP

ATR-FTIR was used to confirm the structure of P3HB, and the spectrum showed distinctive absorption bands that closely matched the published spectral characteristics of pure P3HB (Bayarı and Severcan 2005; Izumi and Temperini 2010). The strongest stretching vibration was observed at 1724 cm^−1^, corresponding to the ester carbonyl group (C = O), a key indicator of the polyester backbone in P3HB (Fig. 4). This peak suggests the presence of well-formed ester bonds, consistent with the expected structure of P3HB. Moreover, the absence of broadening or displacement of the C = O peak indicates a high degree of crystallinity in the polymer. Additional characteristic peaks include the asymmetric and symmetric C–H stretching vibrations of the methylene (CH₂) groups, observed at 2971 cm^−1^ and 2933 cm^−1^, respectively. These peaks further support the presence of an aliphatic hydrocarbon chain typical of P3HB. The band at 1378 cm^−1^ corresponds to the symmetric bending vibration of the terminal methyl (CH₃) groups. Overlapping bands associated with C–O and C–O–C stretching vibrations, characteristic of ester functional groups in the polymer backbone, were also detected in the range of 979 to 1280 cm^−1^.

Thermal analysis was performed to determine the melting temperature and the enthalpy involved in the melting of P3HB. The calorific requirement of the process was calculated using Eq. (5):

5 $$Enthalpy (\Delta H)={\int }_{T1}^{T2}CpdT$$

All biopolymer samples exhibited a broad endothermic inflection during melting (Table 3), indicating the presence of crystalline and amorphous regions. Additionally, the thermal degradation of P3HB under a nitrogen atmosphere was analyzed using TGA. The results showed that thermal degradation occurred between 248 °C and 279 °C, consistent with the thermal stability typically reported for P3HB polymers. Equation (6) illustrates how the melting enthalpy of 100% crystalline P3HB (as reported in the literature) and the melting enthalpy obtained from the thermogram were used to calculate the sample’s degree of crystallinity.

**Table 3:** Glass transition temperature, melting temperature, and degree of crystallinity (heating rate: 10 °C min^−1^) of P3HB extracted from *Bacillus cereus* and *Azotobacter vinelandii* OP under various conditions

P3HB sample description	Melting temperature (◦C)	Heat melting (J g^− 1^)	Maximum degradation temperature (◦C)	Transition enthalpy ΔH (J g^− 1^)	Crystallinity (%)
*Bacillus cereus*					
100 rpm – 24 h	163.08	63.82	253.19	464.55	43.7
100 rpm – 48 h	162.58	107.50	253.43	454.14	73.6
250 rpm – 24 h	149.92	81.02	248.84	483.46	55.4
250 rpm – 48 h	154.89	63.97	251.37	520.54	43.8
*Azotobacter vinelandii* OP					
100 rpm – 48 h	178.79	97.36	279.59	512.80	66.6
100 rpm – 72 h	177.07	87.43	274.73	541.65	59.8
250 rpm – 48 h	176.52	85.16	263.29	486.55	58.3
250 rpm – 72 h	178.02	59.25	262.72	491.29	40.6

6 $$ \begin{aligned} & \% {\text{Crystallinity}}\;{\text{P3HB}} = \\ & \quad \frac{{{\text{Enthalpy}}\;{\text{of}}\;{\text{fusion}}\;({\text{sample}}\;{\text{P3HB}})}}{{{\text{Enthalpy}}\;{\text{of}}\;{\text{fusion}}\;({\text{forfull}}\;{\text{crystalline}})}} \\ & \quad \times 100 \\ \end{aligned} $$

A completely crystalline P3HB system has an enthalpy of fusion of 146 J g^−1^ (Khamplod et al. 2022). The data in Table 3 highlight the importance of fermentation conditions in determining the thermal and structural properties of P3HB synthesized by *B. cereus* and *A. vinelandii* OP. P3HB produced by *B. cereus* exhibited lower melting temperatures (T_m_), ranging from 149.92 °C to 163.08 °C, with the highest value observed at 100 rpm and 48 h of cultivation. By contrast, *A. vinelandii* OP showed more stable T_m_ values between 176.52 °C and 178.79 °C. A similar pattern was observed in the heat of melting, with *B. cereus* reaching a peak value of 107.50 J g^−1^ under the same conditions, while *A. vinelandii* OP recorded its highest value of 97.36 J g^−1^ at 100 rpm and 48 h. P3HB from *A. vinelandii* OP also exhibited higher maximum degradation temperatures (262.72 °C–279.59 °C) compared with that from *B. cereus* (248.84 °C–253.43 °C), indicating greater thermal stability. Crystallinity varied significantly with strain and cultivation conditions. For *A. vinelandii* OP, maximum crystallinity (66.6%) was observed at 100 rpm and 48 h, decreasing to 40.6% at 250 rpm and 72 h. P3HB from *B. cereus* achieved the highest crystallinity among all samples, reaching 73.6% at 100 rpm and 48 h. These differences were further supported by the analysis of transition enthalpy (ΔH), with *B. cereus* reaching a maximum of 520.54 J g^−1^ (250 rpm, 72 h), while *A. vinelandii* OP reached 541.65 J g^−1^ (100 rpm, 72 h). These findings underscore the need to control agitation speed and fermentation duration to fine-tune the characteristics of P3HB. The comparison provides practical insights for industrial applications, suggesting the use of *B. cereus* for processes that benefit from high crystallinity and energy-efficient processing, and *A. vinelandii* OP for applications requiring higher thermal resistance.

#### Analysis of the P3HB mean molecular weight (MMW)

One of the parameters that determines the physicochemical properties of P3HB is the MMW, along with the material’s elasticity and mechanical strength. This property depends on multiple physiological, genetic, and biochemical characteristics of the producing microorganisms—specifically, on metabolic systems that respond to the physiological state of the bacteria. The MMW values obtained by SEC-HPLC for P3HB produced by *B. cereus* and *A. vinelandii* OP at 100 and 250 rpm are discussed below.

**Fig. 5:**
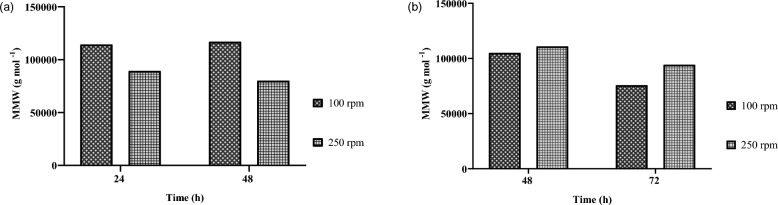
Mean molecular weight of P3HB extracted from ** a** *Bacillus cereus* and ** b** *Azotobacter vinelandii* OP at different cultivation time and agitation rates

The P3HB sample from *B. cereus* at 48 h and 100 rpm corresponds to the highest MMW obtained, calculated at 116,960 g mol^−1^ and is shown in Fig. 5. Interestingly, the cultivation time did not significantly affect the MMW of P3HB in *B. cereus* cultures; however, it can be observed that when the agitation rate was increased from 100 to 250 rpm, the MMW decreased. The conditions studied MMW values between 80,050 g mol^−1^ and 116,960 g mol^−1^, regardless of culture time or agitation rate. At 100 rpm, extending the culture time resulted in only minor MMW increases of 2.3%, while at 250 rpm, a decrease was observed—from 89,264 g mol^−1^ at 24 h to 80,050 g mol^−1^ at 48 h.

Previous studies on the MMW of P3HB in *Bacillus* species are limited. In *B. megaterium* MSBN04, a P3HB MMW of 670,000 g mol^−1^ was reported, with a polydispersity index of 1.71, though this was achieved using solid-state fermentation—a substantially different cultivation method (Sathiyanarayanan et al. 2013). Similar MMW values were obtained with *B. dretensis* BP 17, reaching 115,000 g mol^−1^ in 250-mL shake flasks at 150 rpm using pineapple peel as an alternative carbon source (Penkhrue et al. 2020).

In cultures of *A. vinelandii* OP, the highest P3HB MMW was obtained at an agitation speed of 250 rpm and 48 h of cultivation, reaching a value of 111,000 g mol^−1^, while the lowest MMW was recorded at 100 rpm after 72 h, calculated as 75,805 g mol^−1^. Overall, a decreasing trend in MMW was observed as the cultivation time increased from 48 to 72 h under all conditions tested. MMW decreased by at least 15% with prolonged cultivation. The variability in MMW observed in P3HB from *A. vinelandii* OP cultures is higher than in *B. cereus* cultures. It can be observed that with an increase in the agitation speed from 100 to 250 rpm, the MMW also increased, being 20% higher in 72 h cultures. A dependence on agitation conditions—and therefore on the oxygen transfer rate—can be inferred. The decrease in MMW may be associated with increased enzymatic activity or polymer degradation during the later stages of cultivation. While *A. vinelandii* OP has been extensively studied for its capacity to synthesize P3HB, particularly in bioreactors, there are no reported studies evaluating the molecular weight of the polymer produces by the OP strain under at the flask scale.

In highly agitated cultures, P3HB accumulation may be associated with increased respiration rates and nitrogen limitation, as previously proposed for *A. vinelandii* (Peña et al. 2014). The activation of regulatory systems such as PTSNtr and phbR could explain the observed variations in molecular weight, although these mechanisms were not directly measured in this study. Similarly, under low agitation, reduced oxygen transfer may trigger metabolic adjustments involving the RpoS sigma factor and the PEP-PTS system, as suggested in earlier reports (Deutscher et al. 2006; Peña et al. 2014). These interpretations are speculative but align with known physiological responses to oxygen and nutrient stress. Further studies incorporating molecular analyses would be valuable to confirm these proposed mechanisms.

#### Morphological analysis of cells and P3HB granules by confocal microscopy

**Fig. 6:**
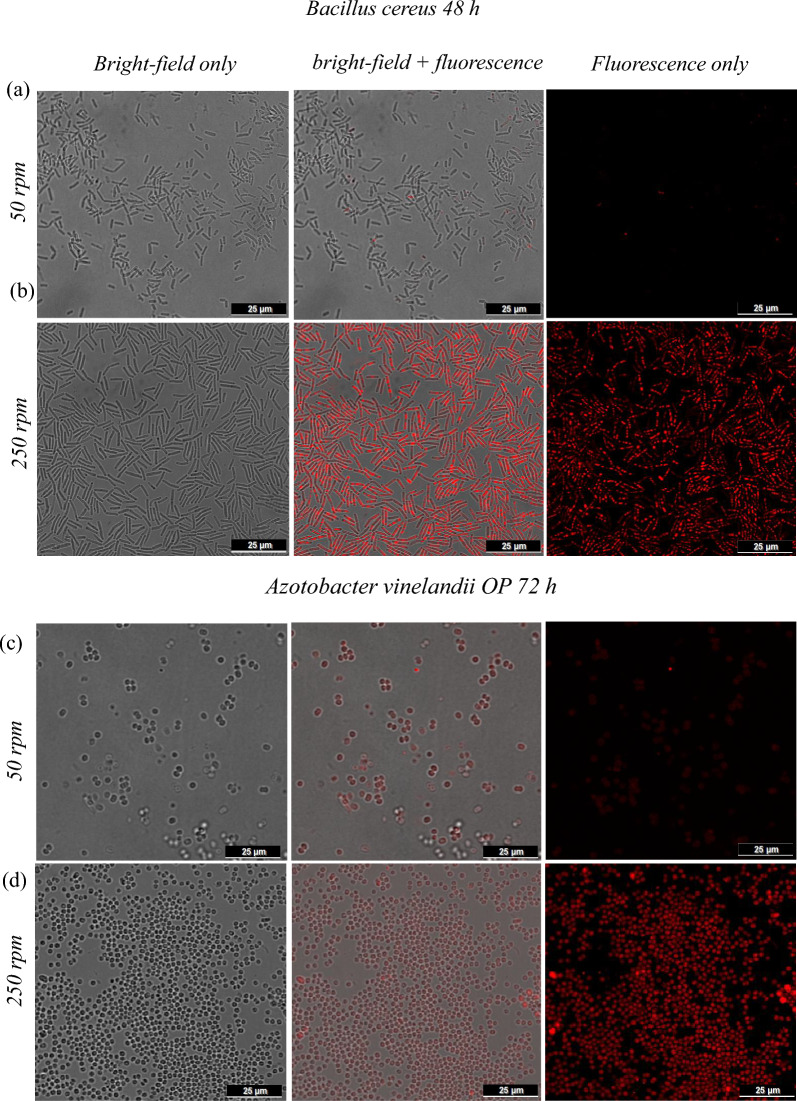
Confocal microscopy images of samples prepared on 1% agarose gel: ** a**, ** b** *Bacillus cereus*; ** c**, ** d** *Azotobacter vinelandii* OP, each at 50 rpm and 250 rpm

Figure 6 shows the fluorescence images obtained by confocal microscopy of *B. cereus* cultures at 48 h and *A. vinelandii* OP at 72 h, under stirring speeds of 50 and 250 rpm. The typical cell morphologies—elongated rods for *B. cereus* and cocci for *A. vinelandii* OP—are clearly visible. In both species, higher fluorescence intensity and a greater number of intracellular granules were observed at 250 rpm, correlating with the higher biomass concentrations achieved under this condition. Additionally, differences in cell size were noted: cells grown at 250 rpm appeared smaller than those cultured at 50 rpm. This observation aligns with previous reports indicating that increased agitation speeds—leading to high shear forces—can reduce bacterial cell size, likely due to enhanced rates of cell division and greater cellular compaction (Lahiri et al. 2021).

## Conclusion

This study demonstrated that agitation rate significantly influences *B. cereus* and *A. vinelandii* cultures, affecting both cell growth and P3HB production, with each species responding differently to the conditions applied at the shake flask scale. While *A. vinelandii* exhibited a higher capacity for P3HB accumulation and greater thermal stability, it also showed greater sensitivity to agitation variations. In contrast, *B. cereus* achieved higher crystallinity indices and greater molecular weights, and its growth and production yields were less affected by agitation. This influence was further analyzed through the analysis of k_L_a, revealing that *A. vinelandii* is more dependent on dissolved oxygen availability. From a bioprocessing standpoint, these findings provide valuable insights into the selection of culture parameters to balance production yields with the physicochemical and mechanical properties of P3HB, depending on the intended application. The results also offer a foundation for future process optimization and scale-up strategies.

This study provides information on the effect of the agitation rate on the production and properties of P3HB at the flask scale, although its results are limited by the lack of pH control and direct measurement of dissolved oxygen (DO). Future experiments at the bioreactor scale, under controlled aeration and stirring conditions, will allow these findings to be validated and explore potential for industrial applicability.

## Data Availability

The data and materials that support the findings of this study are available from the corresponding author upon reasonable request.
